# Deep phenotyping of eyes shut homolog-associated retinopathy based on visual impairment patterns

**DOI:** 10.3389/fopht.2025.1672451

**Published:** 2025-09-08

**Authors:** Daiki Sakai, Yasuhiko Hirami, Satoshi Yokota, Akishi Onishi, Masayo Takahashi, Makoto Nakamura, Yasuo Kurimoto, Akiko Maeda

**Affiliations:** 1 Department of Ophthalmology, Kobe City Eye Hospital, Kobe, Japan; 2 Department of Ophthalmology, Kobe City Medical Center General Hospital, Kobe, Japan; 3 Department of Surgery, Division of Ophthalmology, Kobe University Graduate School of Medicine, Kobe, Japan

**Keywords:** eyes shut homolog (EYS), retinitis pigmentosa, inherited retinal dystrophy, pericentral, genotype-phenotype correlation, phenotyping

## Abstract

**Introduction:**

This study aimed to classify the phenotypes of eyes shut homolog (*EYS*)-associated retinopathy based on visual impairment patterns and investigate their characteristics.

**Methods:**

This retrospective, single-center, cross-sectional study was conducted in 154 patients diagnosed with *EYS*-related retinopathy who underwent genetic testing between December 2017 and July 2023. Phenotyping was performed only in patients who underwent Goldmann perimetry (GP) and Humphrey visual field (HVF) 10–2 testing. Phenotypes were categorized as early, pericentral, typical, and advanced based on peripheral visual field preservation (GP: V-4e isopter extending beyond a 30-degree radius in ≥2 quadrants), central visual field impairment (HVF10-2: ≤20 points with 26 dB sensitivity), and macular impairment (logMAR ≥ 0.2). Genetic and ophthalmological characteristics were compared between the pericentral and typical types.

**Results:**

A total of 39 eyes from 39 patients with *EYS*-associated retinopathy (average age: 48.2 ± 11.9 years, 21 women) were analyzed. Ten pathogenic variants were identified, with the three major variants (p.G843E, p.S1653fs, and p.Y2935X) accounting for a combined allele frequency of 83.3%. The phenotypes were classified as early (n=3), pericentral (n=18), typical (n=9), and advanced (n=9). No significant differences were observed between the pericentral and typical types in terms of the presence of major variants or biallelic null variants. Age and age at onset also did not differ significantly. However, macular impairment was significantly more frequent in the pericentral type (61.8%) than in the typical type (11.1%) (P = 0.014).

**Discussion:**

In *EYS*-associated retinopathy, the pericentral type is considered a common phenotype, although its correlation with the genotype remains unclear. Despite preserved peripheral vision, careful monitoring is warranted due to the risk of macular impairment.

## 1 Introduction

Eyes shut homolog (*EYS*) is a major causative gene of autosomal recessive retinitis pigmentosa (RP) (1), particularly prevalent in Asian countries, including Japan (2, 3). *EYS*-associated retinopathy presents with marked phenotypic heterogeneity, including typical RP, pericentral-type RP, cone-rod dystrophy, and even macular degeneration (4). Despite this variability, a consistent genotype-phenotype correlation has not yet been established. Pericentral-type RP is generally considered an uncommon subtype across various genotypes rather than a distinct clinical entity (5, 6). However, in *EYS*-associated retinopathy, the pericentral type has been recognized as a common phenotype that could account for more than half of the cases (7). Given its distinct pattern of progression and visual impairment compared with the typical type, separate evaluation of these phenotypes may help clarify potential genotype-phenotype correlations. Although diagnostic criteria for pericentral-type RP remain unstandardized, it is commonly defined by early central visual field impairment with relative preservation of the peripheral vision. This study aimed to classify the phenotypes of *EYS*-associated retinopathy into pericentral and typical types based on novel criteria focused on visual field patterns and investigate their clinical and genetic characteristics.

## 2 Methods

### 2.1 Study design

This study was approved by the Medical Ethics Committee of Kobe City Medical Center General Hospital (Kobe, Japan) and was conducted in accordance with the principles of the Declaration of Helsinki. Written informed consent was obtained from all patients prior to genetic testing.

### 2.2 Patients

Medical records of 154 patients with genetically confirmed *EYS*-associated retinopathy who underwent genetic testing at Kobe City Eye Hospital between December 2017 and August 2023 were reviewed. Only patients with available Goldmann perimetry and Humphrey visual field (HVF) 10–2 testing results were included in the study. For patients with multiple visits, the most recent examination results were analyzed. Patients with poor HVF10–2 reliability (fixation loss: >20%, false-positive rate: >15%, or false-negative result: >30%) (three eyes from three patients), coexisting conditions affecting retinal sensitivity (pathological myopia: two eyes from one patient; myopic choroidal neovascularization: one eye from one patient), and previous invasive treatments other than cataract surgery (retinal organoid sheet transplantation: one eye in one patient) were excluded. When both eyes met the inclusion criteria (n = 36), one eye was randomly selected for analysis.

### 2.3 Visual field

Goldmann perimetry (Haag-Streit, Bern, Germany) was performed as a kinetic visual field test. Meanwhile, the HVF 10–2 test using the Swedish Interactive Thresholding Algorithm standard program (Humphrey Field Analyzer; Carl Zeiss Meditec, Dublin, CA, USA) was performed as a static visual field test.

### 2.4 Visual acuity

The best-corrected visual acuity (BCVA) was assessed using Landolt C-charts and converted to the logarithm of the minimum angle of resolution (logMAR) equivalents for statistical analysis. Extremely low visual acuity was assigned logMAR values of 2.0 for counting fingers, 2.3 for hand motion (8), and 2.7 for light perception (9).

### 2.5 Electroretinogram

Full-field electroretinograms (ERGs) were obtained using the LE-3000 system (Tomey, Aichi, Japan) in accordance with the standards of the International Society for Clinical Electrophysiology of Vision (10).

### 2.6 Optical coherence tomography

Cross-sectional OCT images were obtained along the horizontal and vertical meridians through the fovea using Spectralis (Heidelberg Engineering). One observer (D.S.) measured the ellipsoid zone (EZ) width using the “caliper” function of the Heidelberg instrument. The average EZ width was acquired for each patient by averaging the horizontal and vertical EZ widths.

### 2.7 Genetic diagnosis

Genetic testing was performed at Kobe City Eye Hospital between December 2017 and August 2023 using next-generation sequencing. Panels consisting of 39 were used from 2017 to 2019 (2), while 50-gene panels were employed from 2019 onward (11). Variant interpretation and molecular diagnosis were determined through multidisciplinary discussion involving ophthalmologists, clinical geneticists, optometrists, nurses, researchers, and genetic counselors (2). The evaluation was performed with reference to the criteria and guidelines established by the American College of Medical Genetics and Genomics and the Association for Molecular Pathology (12).

### 2.8 Phenotyping of *EYS*-associated retinopathy

**Figure 1:**
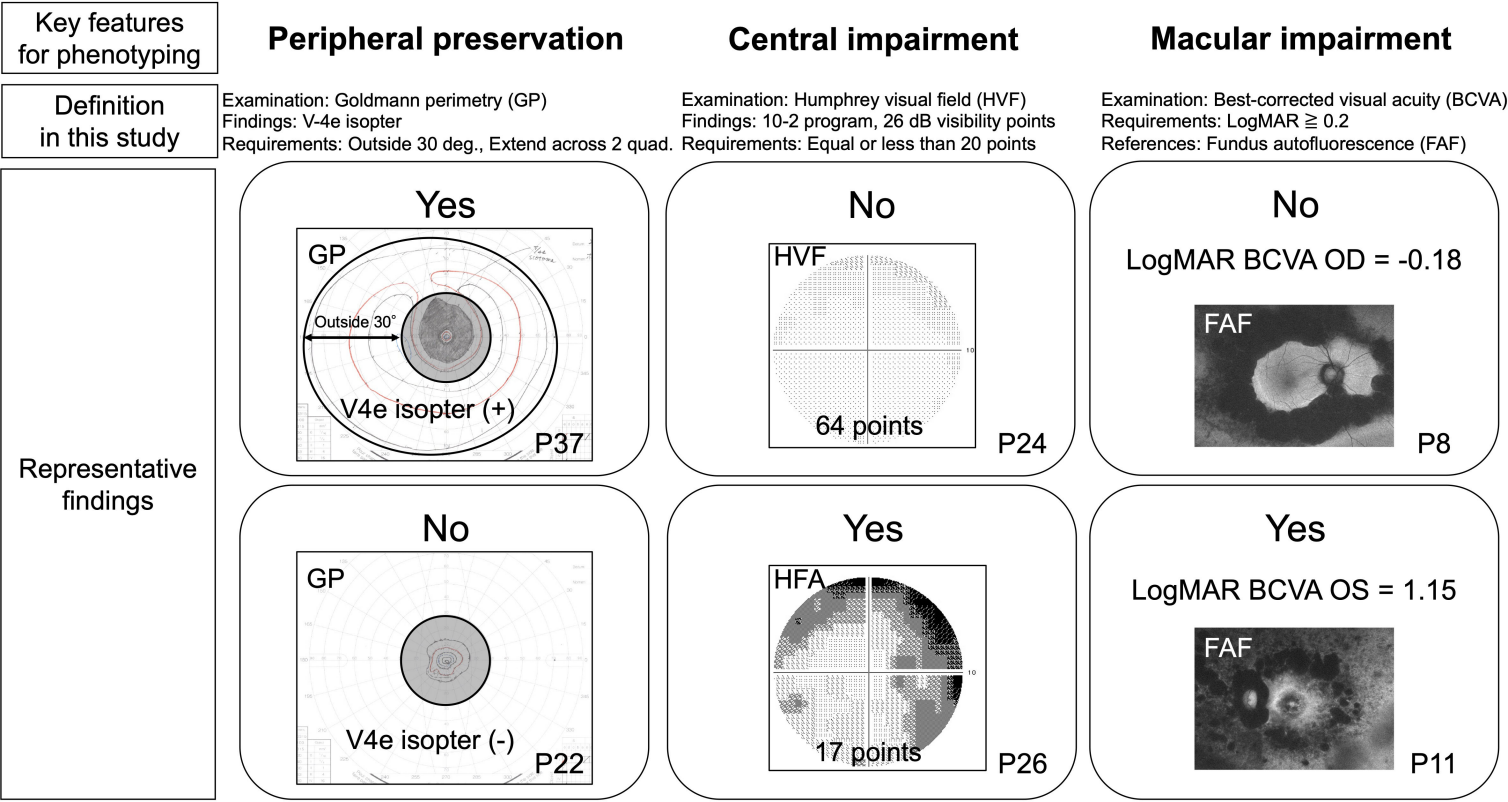
Definition of visual impairment features used for phenotyping *EYS*-associated retinopathy. Patient numbers for each image are indicated with the symbol ‘P’.

Phenotyping was performed based on visual impairment patterns, aimed at distinguishing between pericentral and typical types. The exploratory criteria used in this study consisted of three key features: peripheral preservation, central impairment, and macular impairment (**Figure 1**). Peripheral visual field preservation was identified when the V-4e isopter of Goldmann perimetry extended beyond a 30-degree radius in more than two quadrants (covering at least over 45°angle). The cut-off values for defining central and macular impairments are supported by the criteria for visual impairment certification under the Act on Welfare of Physically Disabled Persons, which is widely accepted in clinical practice in Japan (13). For central impairment, we used a cut-off value of 20 visibility points on the HVF 10-2, corresponding to the criteria for severe visual field impairment (equivalent to Grade 2 physical disability). For macular impairment, we used the visual acuity criterion for impairment certification of LogMAR ≥0.2. Our primary concept was to delineate peripheral and central visual fields, to identify notable central visual field impairment, and to detect macular impairment at an early stage. Phenotypes were classified into four groups based on a combination of these three features. Patients with preserved peripheral visual fields and no evidence of central or macular impairment were classified as having early disease. Those with central visual field impairment and preserved peripheral visual fields were categorized as pericentral type. Patients lacking peripheral visual field preservation were classified as having the typical type, whereas those with additional central and macular impairments were classified as having advanced disease.

### 2.9 Statistical analyses

The characteristics of the pericentral and typical groups were compared to investigate the distinct features of the two phenotypes. Patients with early and advanced disease were excluded from this analysis, as these groups were considered to represent mixed phenotypic features of the pericentral and typical types. Comparisons between the groups were conducted using the unpaired t-test or Mann–Whitney U test for continuous variables, as appropriate, and the chi-square test for categorical variables. All statistical analyses were performed using the SPSS software version 28 (SPSS Inc., Chicago, IL, USA). The significance level for all tests was set at a P value of <0.05.

## 3 Results

This study included 39 eyes from 39 patients with *EYS*-associated retinopathy. The mean (standard deviation) age was 48.2 (11.9) years (range, 29–77), and 21 patients were women. The demographic and clinical characteristics of the participants are presented in **Table 1**. Ten pathogenic variants of the *EYS* gene were identified in this cohort, with three major variants—p.G843E, p.S1653fs, and p.Y2935X—accounting for 83.3% of the allele frequency (**Table 2**).

**Table 1:** Demographic and clinical characteristics of the study participants.

No.	Age	Sex	Genotype				Phenotype											Diagnosis
			Allele1(c.)	Allele1(p.)	Allele2(c.)	Allele2(p.)	Age of onset	Symptoms	GP	HVF10-2			VA		ERG		OCT	
									Peripheral preservation	MD	Score	Central impairment	BCVA (decimal)	Macular impairment	DA 0.01 ERG B-wave (μV)	LA 30Hz ERG (μV)	Average EZ width (μm)	
1	40	Male	c.2528G>A	p.Gly843Glu	c.4957dupA	p.Ser1653fs	27	Night blindness	Yes	-21.91	0	Yes	0.5	Yes			235	Pericentral RP with MD
2	77	Male	c.4957dupA	p.Ser1653fs	c.6557G>A	p.Gly2186Glu	30	Night blindness	No	-31.22	0	Yes	0.4	Yes			631.5	Advanced disease
3	36	Female	c.2528G>A	p.Gly843Glu	c.4957dupA	p.Ser1653fs	15	Night blindness	Yes	-11.82	11	Yes	1.2	No			1922	Pericentral RP without MD
4	50	Male	c.4957dupA	p.Ser1653fs	c.8805C>A	p.Tyr2935*	35	Night blindness	Yes	-34.36	0	Yes	0.06	Yes	66	10.8	0	CRD
5	40	Male	c.2528G>A	p.Gly843Glu	c.4957dupA	p.Ser1653fs	18	Night blindness	Yes	-28.96	12	Yes	1.2	No			1101.5	Pericentral RP without MD
6	69	Female	c.4957dupA	p.Ser1653fs	c.4957dupA	p.Ser1653fs	20	Visual field constriction	No	-31.41	1	Yes	HM	Yes	ext.	16.0	0	Advanced disease
7	39	Male	c.2528G>A	p.Gly843Glu	c.8805C>A	p.Tyr2935*	10	Night blindness	No	-27.77	4	Yes	1	No			1003.5	Typical RP without MD
8	55	Male	c.2528G>A	p.Gly843Glu	c.2528G>A	p.Gly843Glu	30	Visual field constriction	Yes	-1.51	64	No	1.5	No	39.25	26.0	6149	Early disease
9	40	Female	c.2528G>A	p.Gly843Glu	c.8805C>A	p.Tyr2935*	16	Night blindness	No	-14.57	16	Yes	1.2	No	ext.	12.8	2359	Typical RP without MD
10	53	Male	c.1211dup	p.Asn404fs	c.4957dupA	p.Ser1653fs	36	Night blindness	Yes	-34.42	0	Yes	1	No			425	Pericentral RP without MD
11	70	Female	c.2528G>A	p.Gly843Glu	c.4957dupA	p.Ser1653fs	40	Night blindness	Yes	-23.32	1	Yes	0.07	Yes			0	Pericentral RP with MD
12	50	Female	c.2528G>A	p.Gly843Glu	c.8805C>A	p.Tyr2935*	10	Night blindness	Yes	-17.89	0	Yes	0.6	Yes			349.5	Pericentral RP with MD
13	50	Female	c.2528G>A	p.Gly843Glu	c.4957dupA	p.Ser1653fs	39	Night blindness	No	-14.78	27	No	0.5	Yes			3368	Typical RP with MD
14	39	Female	c.2528G>A	p.Gly843Glu	c.2528G>A	p.Gly843Glu	20	Night blindness	Yes	-11.48	33	No	1.5	No	ext.	6.0	3322	Early disease
15	39	Male	c.4957dupA	p.Ser1653fs	c.8805C>A	p.Tyr2935*	20	Night blindness	No	-13.98	10	Yes	0.6	Yes			1217.5	Advanced disease
16	39	Female	c.2528G>A	p.Gly843Glu	c.8196_8200delCTTTC	p.Phe2733fs	20	Night blindness	Yes	-34.91	0	Yes	0.3	Yes			106	Pericentral RP with MD
17	60	Male	c.632G>A	p.Cys211Tyr	c.2528G>A	p.Gly843Glu	35	Night blindness	Yes	-28.39	4	Yes	1	No			817	Pericentral RP without MD
18	30	Female	c.2528G>A	p.Gly843Glu	c.2528G>A	p.Gly843Glu	11	Night blindness	No	-24.9	0	Yes	0.2	Yes			378	Advanced disease
19	56	Female	c.2528G>A	p.Gly843Glu	c.4957dupA	p.Ser1653fs	NA		Yes	-33.82	0	Yes	0.03	Yes			0	Pericentral RP with MD
20	30	Male	c.6557G>A	p.Gly2186Glu	c.6563T>C	p.Ile2188Thr	18	Night blindness	Yes	-27.49	4	Yes	0.7	No			1038	Pericentral RP without MD
21	50	Female	c.6563T>C	p.Ile2188Thr	c.8805C>A	p.Tyr2935*	45	Night blindness	Yes	-22.52	0	Yes	0.3	Yes			587.5	Pericentral RP with MD
22	51	Female	c.2528G>A	p.Gly843Glu	c.8805C>A	p.Tyr2935*	20	Night blindness	No	-7.83	26	No	0.9	No			3014	Typical RP without MD
23	70	Female	c.525_527del	p.Glu176del	c.4957dupA	p.Ser1653fs	12	Night blindness	No	-33.58	0	Yes	HM	Yes			0	Advanced disease
24	48	Female	c.2528G>A	p.Gly843Glu	c.2528G>A	p.Gly843Glu	20	Night blindness	Yes	-1.34	64	No	1.2	No			5977.5	Early disease
25	51	Male	c.7919G>A	p.Trp2640*	c.8805C>A	p.Tyr2935*	20	Night blindness	No	-19.17	5	Yes	0.7	No			1247	Typical RP without MD
26	36	Female	c.4957dupA	p.Ser1653fs	c.4957dupA	p.Ser1653fs	NA		No	-14.26	17	Yes	1.2	No			4787	Typical RP without MD
27	39	Male	c.525_527del	p.Glu176del	c.4957dupA	p.Ser1653fs	31	Night blindness	No	-29.07	1	Yes	0.2	Yes			697.5	Advanced disease
28	39	Female	c.4957dupA	p.Ser1653fs	c.8805C>A	p.Tyr2935*	18	Night blindness	Yes	-21.1	13	Yes	1.2	No			1881.5	Pericentral RP without MD
29	66	Male	c.2528G>A	p.Gly843Glu	c.4957dupA	p.Ser1653fs	30	Night blindness	No	-18.1	14	Yes	1	No			3455	Typical RP without MD
30	53	Female	c.4957dupA	p.Ser1653fs	c.8805C>A	p.Tyr2935*	10	Night blindness	No	-33.25	0	Yes	0.08	Yes			0	Advanced disease
31	57	Male	c.4957dupA	p.Ser1653fs	c.8805C>A	p.Tyr2935*	20	Night blindness	Yes	-17.77	2	Yes	0.4	Yes			364	Pericentral RP with MD
32	57	Female	c.2528G>A	p.Gly843Glu	c.8805C>A	p.Tyr2935*	16	Night blindness	No	-32.85	0	Yes	0.03	Yes			0	Advanced disease
33	45	Female	c.4957dupA	p.Ser1653fs	c.8805C>A	p.Tyr2935*	30	Night blindness	No	-5.95	53	No	1	No			6981.5	Typical RP without MD
34	48	Male	c.7919G>A	p.Trp2640*	c.7919G>A	p.Trp2640*	20	Night blindness	Yes	-28.62	0	Yes	0.5	Yes			278.5	Pericentral RP with MD
35	51	Male	c.4957dupA	p.Ser1653fs	c.8805C>A	p.Tyr2935*	25	Night blindness	No	-34.99	0	Yes	HM	Yes			0	Advanced disease
36	39	Female	c.2528G>A	p.Gly843Glu	c.2528G>A	p.Gly843Glu	35	Visual field constriction	Yes	-17.32	11	Yes	1	No	110.75	59.0	5224	Pericentral RP without MD
37	56	Female	c.2528G>A	p.Gly843Glu	c.4957dupA	p.Ser1653fs	47	Color vision abnormaiy	Yes	-33.23	0	Yes	0.5	Yes	71.5	27.5	454.5	CRD
38	34	Male	c.2528G>A	p.Gly843Glu	c.4957dupA	p.Ser1653fs	13	Night blindness	No	-25.05	1	Yes	0.7	No			868	Typical RP without MD
39	29	Male	c.4957dupA	p.Ser1653fs	c.7919G>A	p.Trp2640*	11	Night blindness	Yes	-35.82	0	Yes	LP	Yes			0	Pericentral RP with MD

GP, Goldmann perimetry; HVF, Humphrey visual field; MD, mean deviation; BCVA, best-corrected visual acuity; ERG, electroretinogram; DA, dark-adapted; LA, light-adapted; OCT, optical coherence tomography; EZ, ellipsoid zone; RP, retinitis pigmentosa; MD, macular degeneration; CRD, cone-rod dystrophy; HM, hand motion; LP, light perception.

**Table 2:** Pathogenic EYS variants identified in the present study.

Nucleotide change	Protein change	Allele count	Homo	Hetero	Allele frequency in this cohort
c.2528G>A	p.Gly843Glu	26	5	16	33.33%
c.4957dupA	p.Ser1653fs	25	2	21	32.05%
c.8805C>A	p.Tyr2935*	14		4	17.95%
c.7919G>A	p.Trp2640*	4		4	5.13%
c.6557G>A	p.Gly2186Glu	2		2	2.56%
c.525_527del	p.Glu176del	2		2	2.56%
c.6563T>C	p.Ile2188Thr	2		7	2.56%
c.1211dup	p.Asn404Lysfs*3	1		1	1.28%
c.632G>A	p.Cys211Tyr	1		1	1.28%
c.8196_8200delCTTTC	p.Phe2733fs	1		1	1.28%

**Figure 2:**
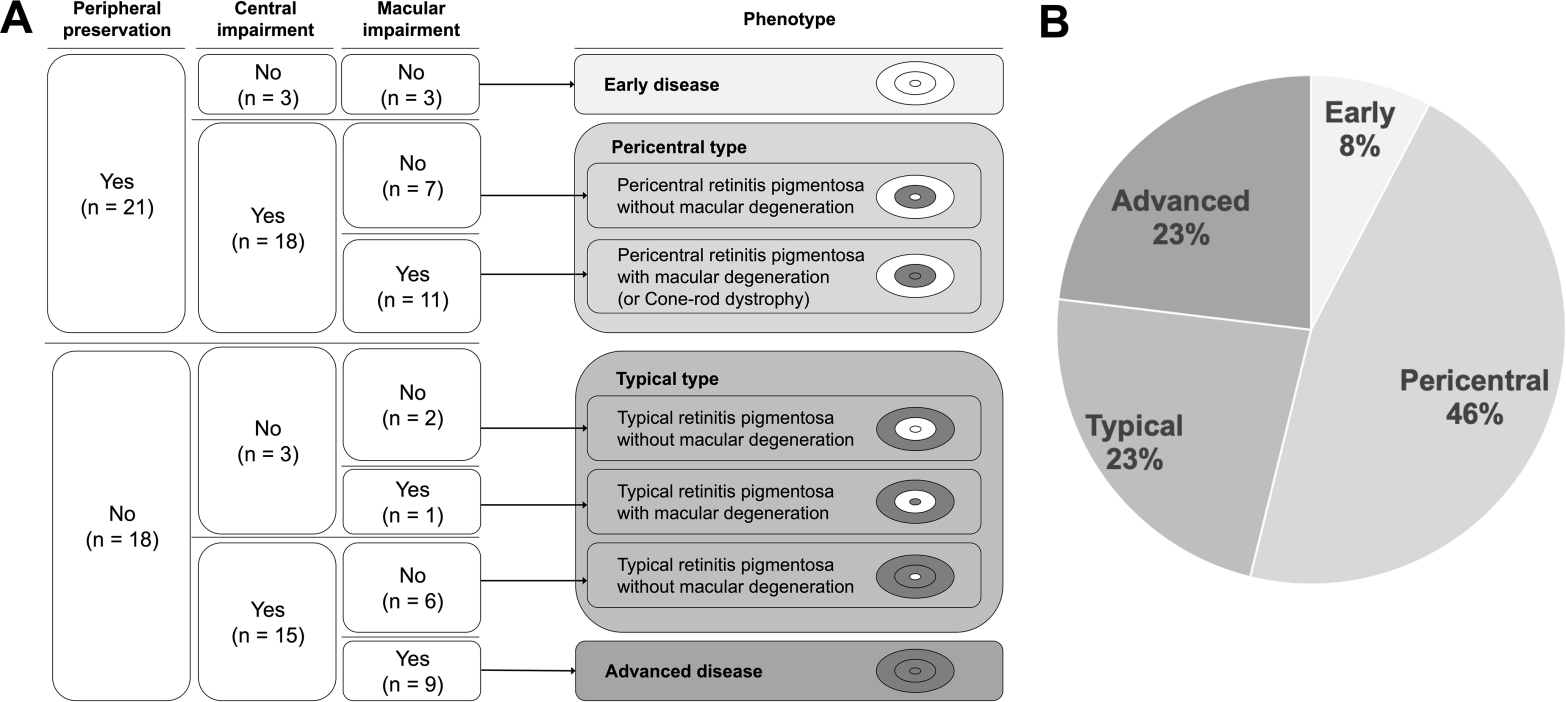
**(A)** Structured criteria for classifying patients with *EYS*-associated retinopathy into four phenotypes: early disease, pericentral type, typical type, and advanced disease. **(B)** Distribution of the four phenotypic groups in this cohort.

**Figure 3:**
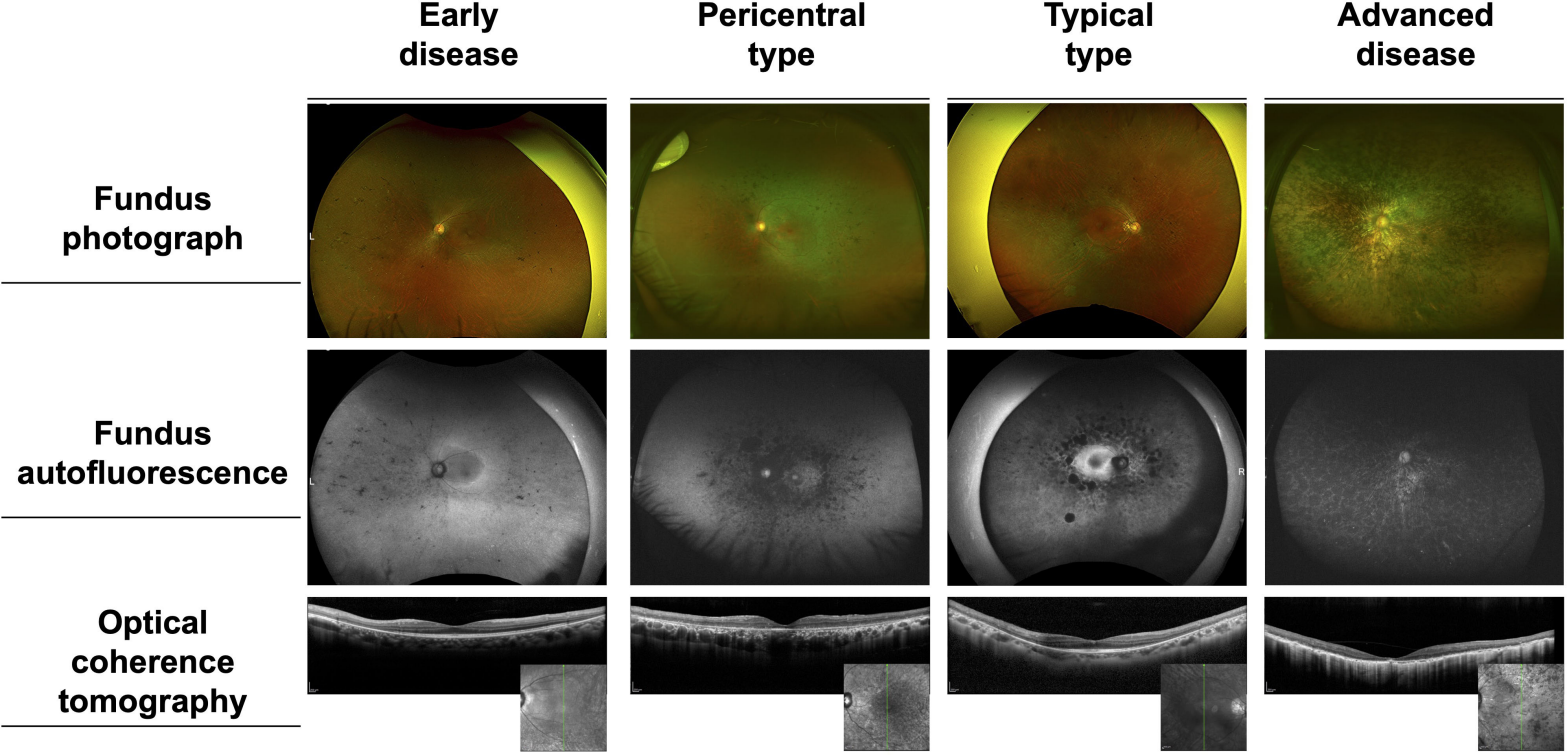
Phenotypic appearances of the four subtypes of *EYS*-associated retinopathy.

The phenotypes were classified as early disease (three patients, 8%), pericentral type (18 patients, 46%), typical type (nine patients, 23%), and advanced disease (nine patients, 23%) (**Figure 2**). A representative phenotypic appearance for each of the four types is shown in **Figure 3**. Among the 39 participants, 21 exhibited preserved peripheral visual fields. Of these, three patients without central visual field or macular impairment were classified as having early disease. The remaining 18 patients with central visual field impairment were identified as having pericentral type. Within this group, 11 patients also presented with macular impairment and were diagnosed with pericentral RP accompanied by macular degeneration. In this subgroup, two patients (Patient No.4 and 37) with available ERG data were further diagnosed with cone-rod dystrophy based on reduced light-adapted 30 Hz ERG responses and preserved dark-adapted 0.01 ERG. Eighteen patients without preserved peripheral visual fields were stratified into the typical group, except for nine patients who exhibited both central visual field and macular impairments and were categorized as having advanced disease. The phenotypic origin of advanced disease in these patients—whether pericentral or typical— could not be determined.

A comparison between the pericentral and typical groups is presented in **Table 3**. No significant differences were observed in age or sex distribution. With regard to genotype, the prevalence of the three major variants—p.G843E, p.S1653fs, and p.Y2935X—did not differ significantly between the groups. Similarly, no differences were found in the prevalence of biallelic truncation variants. Significant differences were observed in the results of the Goldmann perimetry and HVF10–2 tests between the pericentral and typical groups. However, these findings were anticipated, as they were included in the criteria used to define the phenotypes. Although the age of onset did not differ significantly between the groups, the frequency of macular impairment was significantly higher in the pericentral group (61.1%) compared with the typical type (11.1%) (P = 0.014). The logMAR BCVA was worse in the pericentral group, although the difference was not significant. The average EZ width was significantly shorter in the pericentral group compared with that in the typical group (P < 0.001).

**Table 3:** Comparison of genotypic and phenotypic characteristics between the pericentral and typical groups.

	Pericentral	Typical	P
	n = 18	n = 9	
Age (years), mean ± SD	46.8 ± 11.0	45.8 ± 10.0	0.820
Sex (female/male)	9/9	5/4	0.785
Genotype			
Harboring at least one c.2528G>A (p.Gly843Glu) variant	10/18 (55.6%)	6/9 (66.7%)	0.580
Harboring at least one c.4957dupA (p.Ser1653fs) variant	11/18 (61.1%)	5/9 (55.6%)	0.782
Harboring at least one c.8805C>A (p.Tyr2935X) variant	5/18 (27.8%)	5/9 (55.6%)	0.159
Biallelic truncation variants	6/18 (33.3%)	3/9 (33.3%)	1.000
Phenotype			
Age of onset (years), mean ± SD	26.5 ± 11.8	22.3 ± 9.9	0.392
LogMAR BCVA, mean ± SD	0.50 ± 0.72	0.06 ± 0.13	0.079
Macular impairment, n (%) (LogMAR≧0.2)	11/18 (61.1%)	1/9 (11.1%)	0.014*
Humphrey visual field 10–2 testing			
Mean deviation (dB), mean ± SD	−26.3 ± 7.4	−16.4 ± 7.1	0.003*
Central visual field visibility points (CVFVP), mean ± SD	3.2 ± 4.9	18.1 ± 16.0	0.001*
Central visual field impairment, n (%) (CVFVP ≤ 20)	18/18 (100%)	6/9 (66.7%)	0.009*
Goldmann perimetry			
Peripheral preservation, n (%)	18/18 (100%)	0/9 (0%)	<0.001*
Optical coherence tomography			
Average EZ width (μm), median (IQR)	394.5 (974.4)	3014 (2996)	<0.001*

SD, standard deviation; BCVA, best-corrected visual acuity; EZ, ellipsoid zone; IQR, interquartile range.

*Significant at p <0.05.

## 4 Discussion

In this study, a phenotyping analysis of patients with *EYS*-associated retinopathy was conducted using novel criteria based on patterns of visual impairment. The pericentral-type was identified as a common phenotype, accounting for approximately half of the cases, which is consistent with the findings of a previous study (7). The genotypic and phenotypic characteristics of the pericentral and typical types were compared. However, no genotypic markers were identified that could clearly distinguish these two phenotypes. Macular impairment was more frequent in the pericentral group, suggesting a clinically relevant feature that may warrant careful monitoring in the management of pericentral-type *EYS*-associated retinopathy.

Inherited retinal diseases, including *EYS*-associated retinopathy, were historically considered incurable; however, recent advancements in innovative therapies have marked the beginning of a new era. Among these, retinal gene therapy has emerged as a promising therapeutic approach. Conventional gene delivery methods using adeno-associated viral (AAV) vectors are limited by their cargo capacity and are not readily applicable to large genes, such as *EYS*. However, emerging technologies, such as AAV dual vectors (14) or localized gene editing (15, 16) are expected to overcome these limitations. Additionally, gene-independent therapies, such as stem cell-based retinal cell transplantation (17) and optogenetic therapy (18) may be applicable to non-early-stage *EYS*-associated retinopathy. Modifying chronic retinal inflammation is another promising therapeutic strategy and antioxidant therapy with oral N-acetylcysteine (19) is currently in a Phase 3 clinical trial. To optimize therapeutic selection, a detailed understanding of phenotype characterization is increasingly important. *EYS*-associated retinopathy exhibits considerable phenotypic variability, with a notably high prevalence of the pericentral type. This subtype has been described as initiating in the near periphery in contrast to the mid-peripheral onset typical of classic retinitis pigmentosa. Although pericentral RP is often regarded as a milder phenotype due to preserved peripheral vision, early involvement of the central visual field is commonly observed. In this study, 18 of the 39 patients (46%) were classified as having the pericentral phenotype, characterized by central visual field impairment that corresponds to the second-highest level of visual disability certification in Japan (13). Medical care for these patients should be tailored to address central vision deterioration and the therapeutic strategy should focus on preserving central vision or cone function during the early to mid-stages of the disease, while enhancing peripheral vision at the later stages.

Although the pericentral and typical phenotypes could be clearly distinguished based on the patterns of visual impairment, no genotypic features—including the three major variants (p.G843E, p.S1653fs, and p.Y2935X) or the presence of biallelic truncation variants—were found to delineate these two phenotypes. To date, several potential genotype-phenotype correlations have been proposed in *EYS*-associated retinopathy. For example, biallelic truncation variants (20) or variants located near the C-terminal (7) have been suggested to be associated with more severe visual acuity decline. However, these correlations remain inconclusive, as previous studies have demonstrated that phenotypic heterogeneity persists among patients with similar genotypes (7, 21). The *EYS* gene (MIM *612424) encodes the human ortholog of Drosophila *Eys*/spacemaker, which is believed to maintain the structural integrity of photoreceptors (22). Its molecular functions have been primarily investigated in zebrafish models, which suggest that the *EYS* is localized near the connecting cilium of photoreceptors and functions as an extracellular protein essential for ciliary activity (23). However, the pathogenic mechanisms of *EYS* mutations in the human retina remain unclear, due to the absence of an animal model that accurately recapitulates human *EYS*-associated retinopathy. The establishment of such a model would greatly facilitate future studies. Both this study and previous clinical studies have failed to establish definitive genotype-phenotype correlations to account for the observed phenotypic variability in *EYS*-associated retinopathy. Namely, even with the same genotype, some patients exhibited the pericentral phenotype while others exhibited the typical phenotype, supporting the hypothesis that background modifiers may exist. To our knowledge, specific modifier genes for *EYS*-associated retinopathy have not yet been identified. However, it is supposed that the phenotypic diversity is likely influenced by a broader genetic background, including non-cording region variants (24) or epigenetic factors like promoter hyper methylation (25). Furthermore, environmental factors such as diet and light exposure may influence antioxidant capacity or metabolic status, affecting disease expression, as is common in other forms of retinitis pigmentosa.

Deep phenotyping was performed using structured criteria based on the patterns of visual impairment. However, comprehensive genotyping was limited by the relatively small sample size, which may have precluded the identification of genotype-phenotype correlations. Our inclusion criteria, which required both Goldmann perimetry and HVF 10–2 testing results, led to a small cohort. We found that the EZ width on OCT was significantly shorter in the pericentral type compared to the typical type, consistent with the central impairment assessed by the HVF 10–2 testing. Given that OCT results are readily available, integrating this structural parameter into the classification system as a central impairment assessment could enhance the feasibility of studying a wider patient cohort in future research. Another limitation was the absence of longitudinal follow-up data. Although cross-sectional analysis enabled characterization of the clinical features associated with the pericentral and typical phenotypes, prospective longitudinal studies with larger cohorts are necessary to validate and expand upon these findings. In conclusion, the pericentral type is considered a common phenotype of *EYS*-associated retinopathy. Structured classification based on visual impairment patterns is useful for distinguishing between pericentral and typical phenotypes. Although the pericentral type is marked by preserved peripheral vision, early involvement of the macula is not uncommon and warrants careful clinical monitoring.

## Data Availability

The original contributions presented in the study are included in the article/supplementary material. Further inquiries can be directed to the corresponding author.
